# Effect of composition and thermal history on deformation behavior and cluster connections in model bulk metallic glasses

**DOI:** 10.1038/s41598-022-20938-6

**Published:** 2022-10-12

**Authors:** Nico Neuber, Maryam Sadeghilaridjani, Nandita Ghodki, Oliver Gross, Bastian Adam, Lucas Ruschel, Maximilian Frey, Saideep Muskeri, Malte Blankenburg, Isabella Gallino, Ralf Busch, Sundeep Mukherjee

**Affiliations:** 1 Chair of Metallic Materials, Saarland University, Campus C6.3, 66123 Saarbrücken, Germany; 2 Department of Materials Science and Engineering, University of North Texas, Denton, TX 76203 USA; 3 Amorphous Metal Solutions GmbH, 66424 Homburg, Germany; 4 Deutsches Elektronen-Synchrotron DESY, Notkestr. 85, 22607 Hamburg, Germany

**Keywords:** Glasses, Mechanical properties, Metals and alloys, Condensed-matter physics, Structure of solids and liquids

## Abstract

The compositional dependence and influence of relaxation state on the deformation behavior of a Pt–Pd-based bulk metallic glasses model system was investigated, where platinum is systematically replaced by topologically equivalent palladium atoms. The hardness and modulus increased with rising Pd content as well as by annealing below the glass transition temperature. Decreasing strain-rate sensitivity and increasing serration length are observed in nano indentation with increase in Pd content as well as thermal relaxation. Micro-pillar compression for alloys with different Pt/Pd ratios validated the greater tendency for shear localization and brittle behavior of the Pd-rich alloys. Based on total scattering experiments with synchrotron X-ray radiation, a correlation between the increase in stiffer 3-atom cluster connections and reduction in strain-rate sensitivity, as a measure of ductility, with Pd content and thermal history is suggested.

## Introduction

Bulk metallic glasses (BMGs) are a relatively new class of metallic materials which have attracted considerable attention in structural applications over the past decades due to their outstanding mechanical properties such as high strength, large elastic limit, excellent irradiation, wear, and corrosion resistance in the glassy state and thermoplastic forming ability in the supercooled liquid state^1–4^. However, limited plasticity at room temperature in bulk form has restricted their widespread use^5,6^. Without dislocations and grain boundaries, BMGs show completely different deformation mechanism compared to conventional crystalline alloys^7^. Plastic deformation in metallic glasses tends to occur in the form of highly localized shear bands, which, depending on the mode of loading, may result in catastrophic failure^8,9^. Several different approaches have been introduced to enhance the plasticity of BMGs through altering of their chemistry and processing conditions such as ex-situ and in-situ fabrication of BMG matrix composites (BMGMCs)^10^, metal coating confinement^11^, heat treatment^12^, ion radiation^13^, and increase in Poisson’s ratio^14^. These studies aimed to control and manipulate the processes of shear-band nucleation and propagation. Cold rolling at room temperature has been utilized to increase the intrinsic plasticity of BMGs by introducing microstructural inhomogeneities, leading to nucleation and branching of shear bands upon deformation^15^. Ti-based BMG composites with volume fraction of the glassy phase in the range of 20–70% exhibited ~ 5% tensile ductility, which is comparable to conventional polycrystalline titanium alloys^10^. In another study, the plasticity of Zr-based BMGs was improved with the addition of quasicrystals in the glassy matrix^16^. In case of Nd_60_Al_10_Ni_10_Cu_20−x_Fe_x_ BMGs, compositional adjustments with the addition of Fe changed the deformation behavior from inhomogeneous to homogeneous plastic flow^17^. The effect of strain rate and temperature on deformation behavior of various BMGs has also been reported^18–24^. However, there are few studies on systematic series of interrelated glass-forming alloys^20^ and limited understanding on the effect of chemistry and local atomic structure on deformation behavior of BMGs. This is critical in the rational design of new classes of BMGs with superior mechanical properties.

Glass formation is often limited to a narrow region in compositional space for metallic systems^25^. In the case of Pd-P- and Pt-P-based liquids, the high glass-forming ability (GFA)^26^, similarity of phase diagrams^27,28^, and topological equivalency of Pt and Pd^29,30^ make them model alloys for obtaining a systematic series of interrelated alloys. This idea is further supported by their similar temperature dependence of equilibrium viscosity (fragility) in the deeply supercooled liquid state^31–34^. However, their GFA varies by a factor of four^35,36^ and they have significantly different entropies of fusion, ΔS_f_^31,34,37,38^. The larger ΔS_f_ and more rapidly ascending heat capacity upon cooling for the Pt-P-based liquids stand out from those of the Pd-P-based liquids, indicating different atomic ordering processes upon undercooling^37,38^. The high GFA of Pd-P-based liquids originates from an extremely low driving force for crystallization, whereas the Pt-P-based liquids are stabilized by a high interfacial energy between the liquid and the crystal^33,37^, which points to unique structural differences between the two systems.

Here, the influence of compositionally and thermally induced change in the distribution of dominant polyhedra and the associated variation in connection schemes in Pt–Pd-based BMGs on their deformation behavior is evaluated. Nanoindentation and micro-pillar compression are used for the mechanical characterization^4,39–45^. With gradual replacement of Pt by Pd, significant changes are noted in hardness and modulus, strain rate sensitivity, shear transformation zone volume, and serrated flow behavior. The evolution in mechanical properties is discussed in terms of their internal structure differences. Embrittlement effects, resembling those induced by changes in composition, are observed by annealing below the glass transition temperature (T_g_). Further, synchrotron studies reveal that changes in mechanical properties are mirrored by the structural signatures in terms of the varying connection schemes, providing valuable insights into structure–property correlations in metallic glasses.

## Results

### Serration behavior, hardness, and modulus

**Figure 1:**
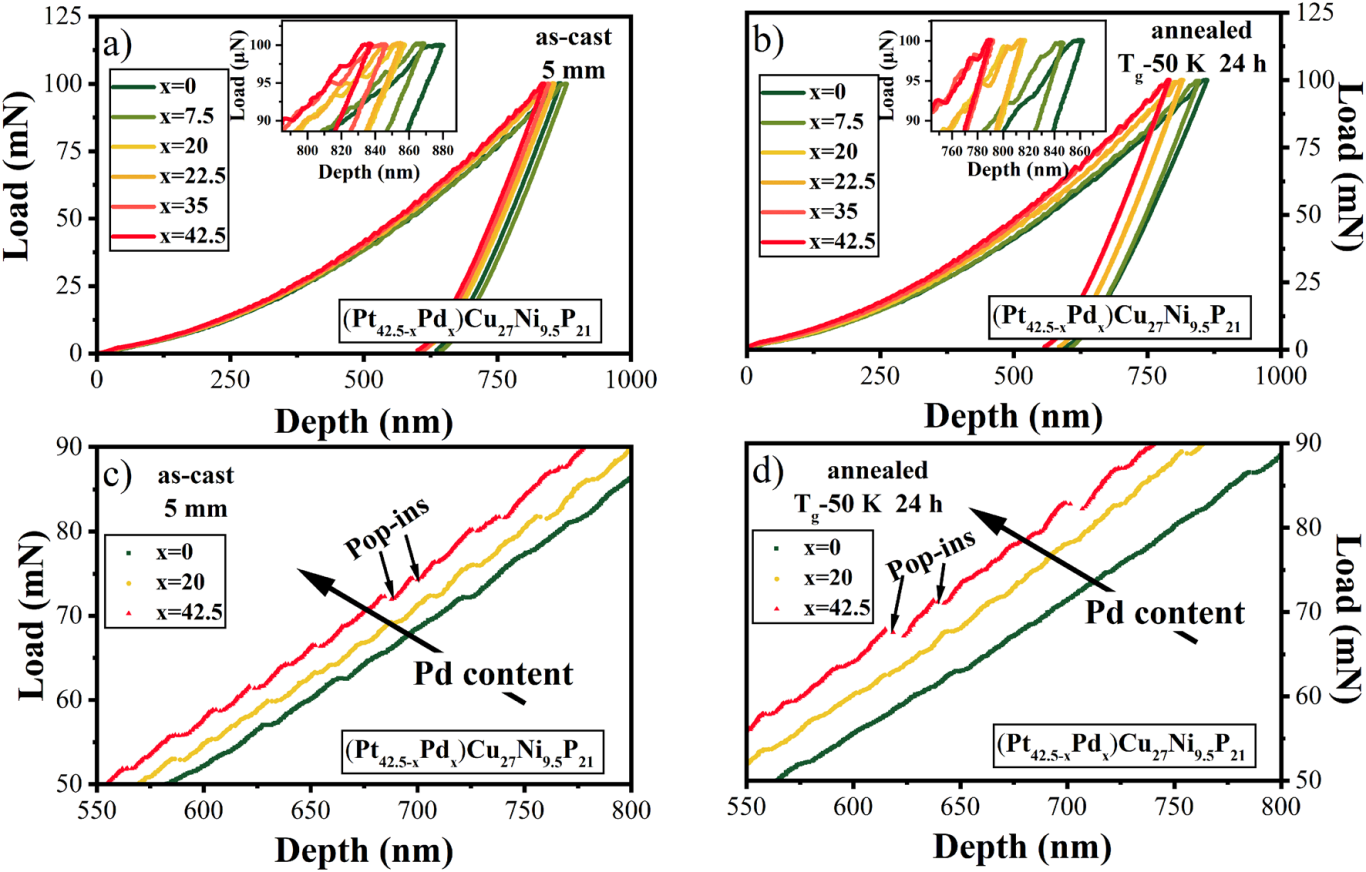
Nano-indentation load-depth plots for Pt_42.5−x_Pd_x_Cu_27_Ni_9.5_P_21_ bulk metallic glasses as a function of Pd content (i.e., x = 0,7.5, 20, 22.5, 35, 42.5); (**a**) in the as-cast state of a 5 mm rod and (**b**) after isothermal annealing at T_g_-50 K for 24 h; The insets in parts (**a**) and (**b**) show the zoomed in view of the loading curves; (**c**) and (**d**) show serrated behavior for Pd_42.5_Cu_27_Ni_9.5_P_21_ (x = 42.5) with large displacement bursts (pop-ins) and relatively smooth curve for Pt_42.5_Cu_27_Ni_9.5_P_21_ (x = 0) in the as-cast and annealed states.

Figure 1a) and b) show the representative nano-indentation load-depth curves for Pt_42.5−x_Pd_x_Cu_27_Ni_9.5_P_21_ amorphous alloys as a function of Pd-content (x = 0–42.5) in the as-cast and annealed states. The reduction in indentation depth *h* with increase in Pd content for both, the as-cast and annealed states (insets of Fig. 1a, b), suggests a rise in hardness value with increase in Pd content. Also, the degree of serrations, which is associated with shear strain accommodation^17,46–48^, increases with Pd content as shown in the zoomed in regions of the loading curves in Figs. 1c, d. The loading curve appears relatively smooth for the Pd-free alloy in contrast to large displacement bursts or “pop-ins” seen for the Pd-rich BMGs^48^. Comparably, a decrease of serrated flow was reported in a prior study with increase in Fe content for Nd_60_Al_10_Ni_10_Cu_20−x_Fe_x_ BMGs^17^.

**Figure 2:**
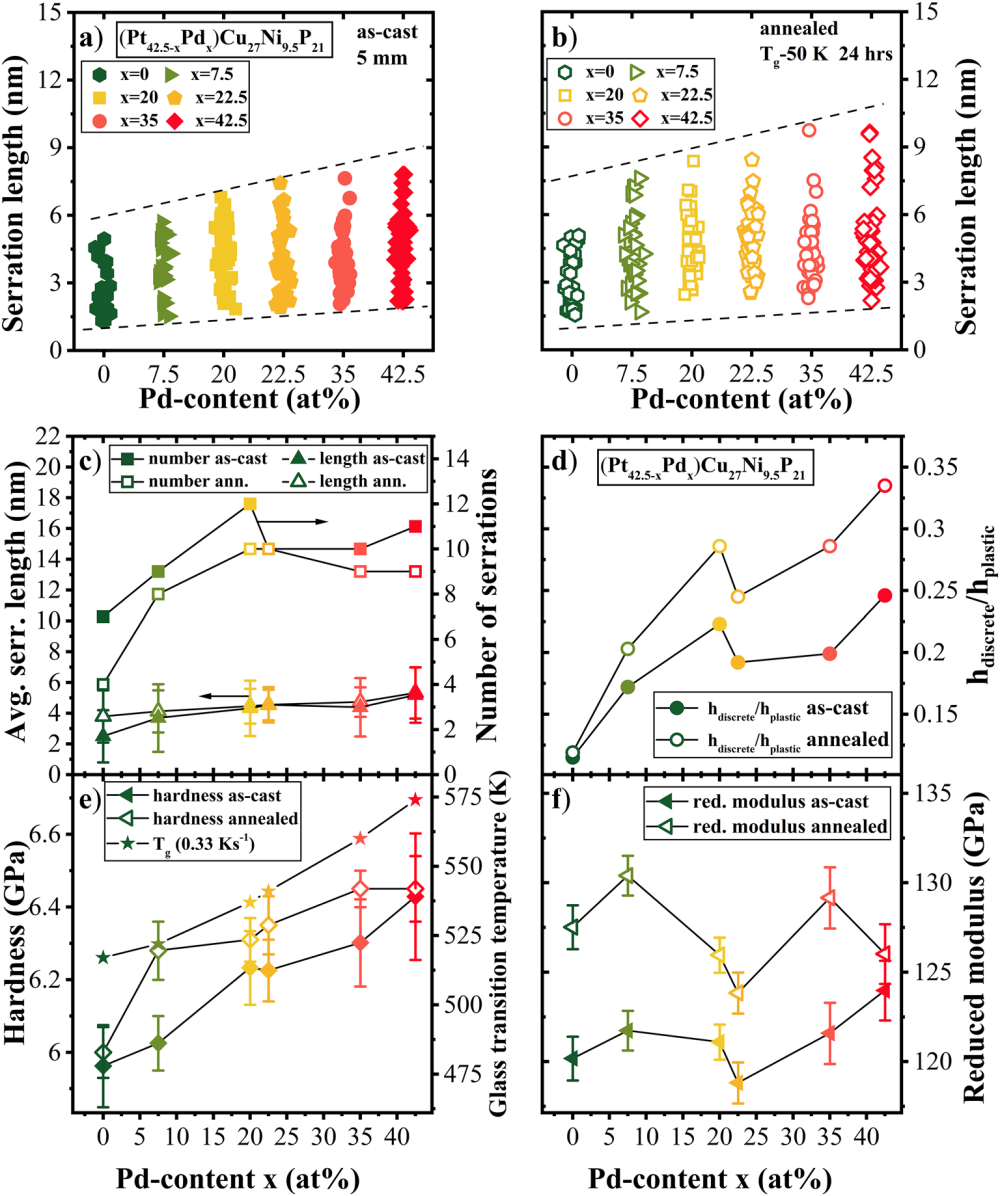
(**a**) and (**b**) Distribution of serration lengths for Pt_42.5−x_Pd_x_Cu_27_Ni_9.5_P_21_ bulk metallic glasses as a function of Pd content (i.e., x = 0, 7.5, 20, 22.5, 35, 42.5) in as-cast and annealed states; (**c**) average serration length and serration frequency, (**d**) plasticity ratio, (**e**) hardness and glass transition temperature, and (**f**) reduced modulus for Pt_42.5−x_Pd_x_Cu_27_Ni_9.5_P_21_ amorphous alloys as a function of Pd content from 0 to 42.5 at.% in the as-cast and annealed state (T_g_-50 K for 24 h). An increase in magnitude and frequency of serration as well as hardness and modulus with increasing Pd content is seen. Annealing of the samples leads to similar increase in hardness and modulus.

For quantitative analysis, the average serration length and serration frequency were calculated from the load–displacement curves. Figure 2a, b show the distribution of displacement bursts in the loading curves for Pt_42.5−x_Pd_x_Cu_27_Ni_9.5_P_21_ amorphous alloys as a function of Pd content from 0 to 42.5 at. % in the (a) as-cast and (b) annealed state. The spread of the minimum and maximum serration length broadens with increase in Pd content. Compared to the as-cast state, the annealed samples show a slightly broader distribution in serration length over the whole compositional range. Figure 2c shows the average serration length of both states summarized together with the number of serrations as a function of Pd content. For the as-cast state, the average serration length increases from 2.5 to 5 nm and the number of serrations increases from 5 to 15 over the load range studied with increase in Pd content from 0 to 42.5 at.%. Annealing of the samples did not lead to a significant change in average serration length or the number of serrations for all the compositions. The discrete plasticity ratio, *h*_discrete_/*h*_plastic_, is depicted in Fig. 2d), showing a similar trend as the number of serrations with change in composition. This parameter helps in determining the contribution of serrated flow on the total plastic deformation. It was estimated from the sum of each individual pop-in (*h*_discrete_ = Σ*h*_pop-in_) divided by the residual indentation depth after releasing the load (*h*_*plastic*_). The discrete plasticity ratio continuously increased with addition of Pd from 0.115 to 0.246 for the as-cast samples and from 0.119 to 0.335 for the annealed samples. In summary, annealing and Pd addition led to higher discrete plasticity ratio and decreased residual indentation depth.

The hardness (*H*) and reduced modulus (*E*) determined from load-depth curves are shown in Fig. 2e, f, respectively. The average hardness increased from ~ 5.9 GPa for Pt_42.5_Pd_0_ (x = 0) to ~ 6.5 GPa for Pt_0_Pd_42.5_, following the same trend as the glass transition, which is also plotted on the right axis of Fig. 2e). Annealing led to a slight increase in hardness, which may be attributed to the reduction in free volume and denser packing. Increase in modulus with higher Pd content and with annealing was observed indicating higher stiffness for alloys with higher Pd content as well as after thermal annealing that leads to relaxation. With decreasing free volume or increasing ordering, the average inter-atomic distance decreases, resulting in increased material stiffness or elastic modulus^48^.

### Strain Rate Sensitivity and Shear Transformation Zone Volume

**Figure 3:**
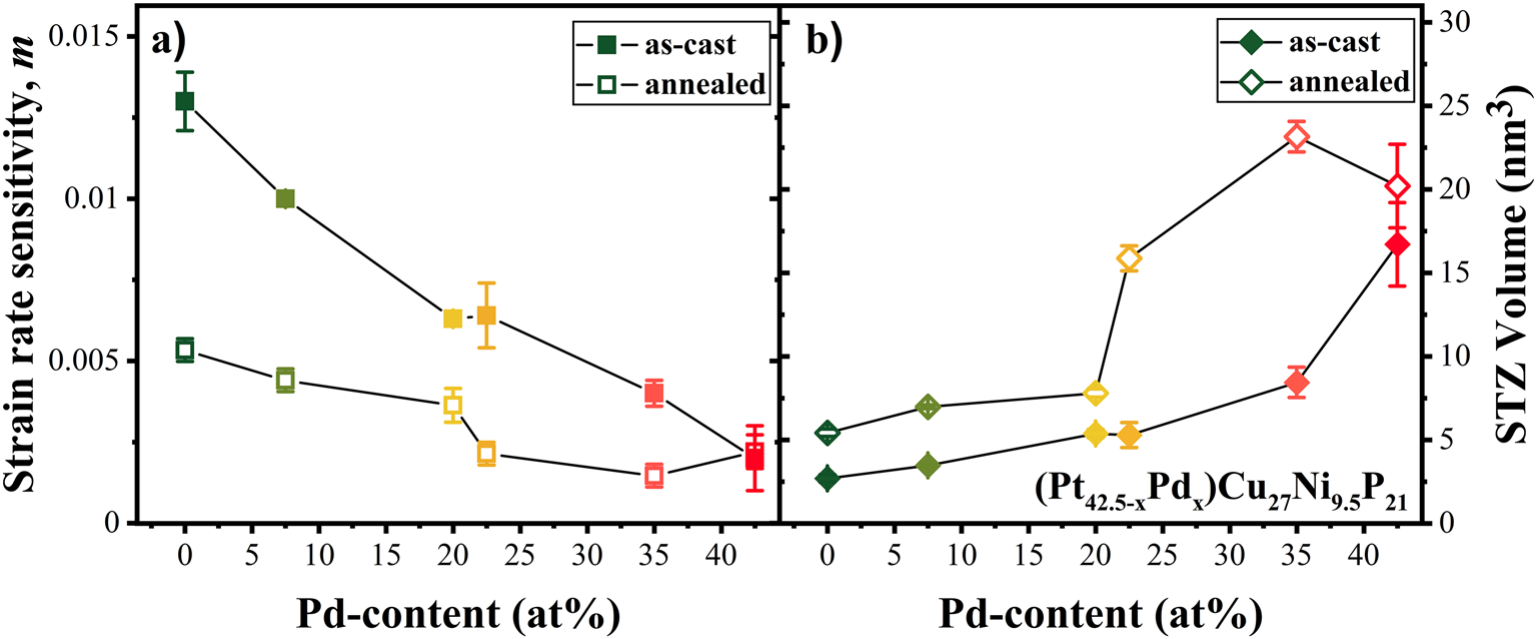
(**a**) Strain rate sensitivity, *m,* as a function of Pd content for the as-cast and annealed state obtained from linear fitting of hardness versus logarithm of strain rate (shown in Supplementary Information Fig. S1); (**b**) STZ volume versus Pd concentration for as-cast and annealed state, showing the lowest STZ volume for Pt_42.5_Pd_0_ alloy supporting its more homogeneous plastic flow.

For all the examined Pt_42.5−x_Pd_x_Cu_27_Ni_9.5_P_21_ amorphous alloys, hardening was observed with increasing applied strain rate, described by a positive strain-rate sensitivity (SRS). The hardness values as a function of strain rate at a depth of 1000 nm for the Pt_42.5−x_Pd_x_Cu_27_Ni_9.5_P_21_ amorphous alloys are shown in Supplementary Information Fig. S1 on a double logarithmic scale. The strain rate sensitivity, *m*, was calculated from the slope of linear fitting ^41^ (see SI Fig. S1) and is reported in Fig. 3a) as a function of Pd content. Similar positive SRS in the range of 0.006–0.036 has been reported for various bulk metallic glasses^21,49–51^ and may be attributed to the delay in shear band activation with increasing strain rate. A higher value of SRS indicates greater resistance to localized plastic deformation and is therefore associated with more ductile deformation behavior^52^. The Pt_42.5_Pd_0_ alloy showed almost an order of magnitude larger *m* value in comparison to the Pt_0_Pd_42.5_ alloy^53^. This is in line with the smooth load-depth curves for the Pt-rich glasses in contrast to more serrated behavior seen for the Pd-rich glasses (Figs. 1 and 2).

Structural relaxation due to thermal annealing led to a decrease in SRS for the entire compositional spread. However, hardness for the Pd-rich glasses was fairly insensitive to shear rate, while a significant drop in SRS of ~ 50% between the annealed and as-cast state was observed for the Pt-rich glasses. Comparing the effect of Pd addition and annealing and using SRS as a measure of ductility, the 24 h annealing at T_g_-50 K for the Pt_42.5_Pd_0_ glass had a similar effect as substitution of ~ 50% of Pt by Pd.

During plastic deformation of a metallic glass, clusters of atoms undergo cooperative shear displacement in response to external applied stress, accommodating the plastic strain over a region known as shear transformation zone (STZ). A shear band is nucleated from the local accumulation of STZs and STZ volume provides estimation of the number of atoms involved in shear transformation in a metallic glass^54^. STZ volume for all the Pt_42.5−x_Pd_x_Cu_27_Ni_9.5_P_21_ BMGs was calculated based on Johnson–Samwer cooperative shearing model (CSM)^21^ and shown in Fig. 3b) for the as-cast and annealed state. The STZ volume of the as-cast BMGs varies from ~ 2.5 to ~ 18 nm^3^, increasing with Pd concentration. The smaller STZ volume for Pt_42.5_Pd_0_ enables the activation of greater number of flow units, leading to the nucleation of more shear bands and promoting more ductile behavior in contrast to Pt_0_Pd_42.5_ with its large STZ volume^6,55^. Similarly, larger STZ volume is seen for the annealed alloys in the range of 6 nm^3^ to 23 nm^3^. Addition of Pd and thermal annealing showed similar effect in terms of increasing STZ volume, with the effect of annealing being more significant on the Pd-rich side of the composition spread.

### Stress–strain and deformation behavior

**Figure 4:**
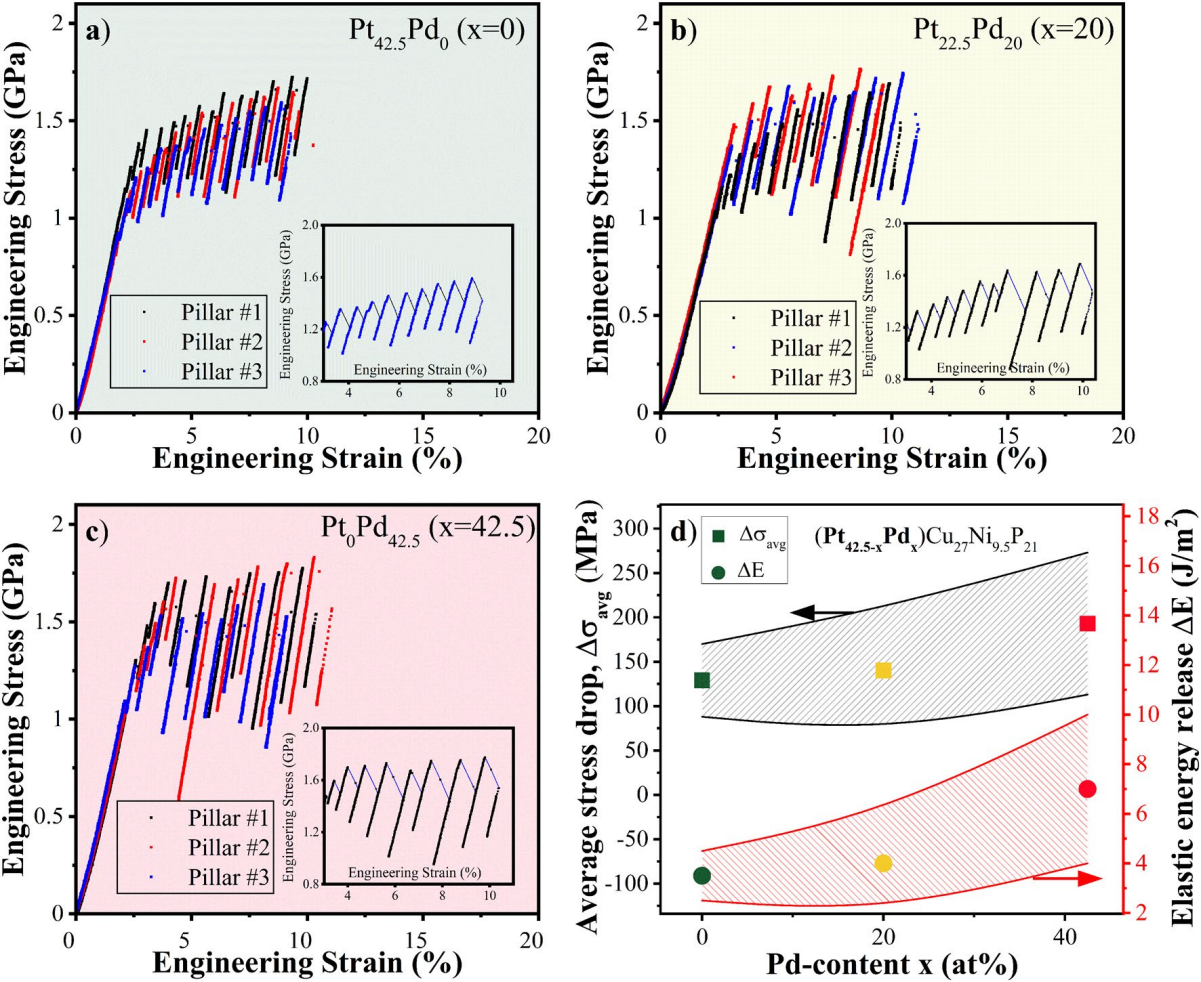
Engineering stress–strain curves for: (**a**) Pt_42.5_Pd_0_, (**b**) Pt_22.5_Pd_20_, and (**c**) Pt_0_Pd_42.5_ alloys in the as-cast state. Three pillars were tested for each alloy as shown. The insets show zoomed-in view of the serrations for each alloy; (**d**) Average value of the magnitude of stress-drop and stored elastic energy as a function of Pd content showing larger stress drop with higher elastic energy released for alloys with higher Pd content.

**Figure 5:**
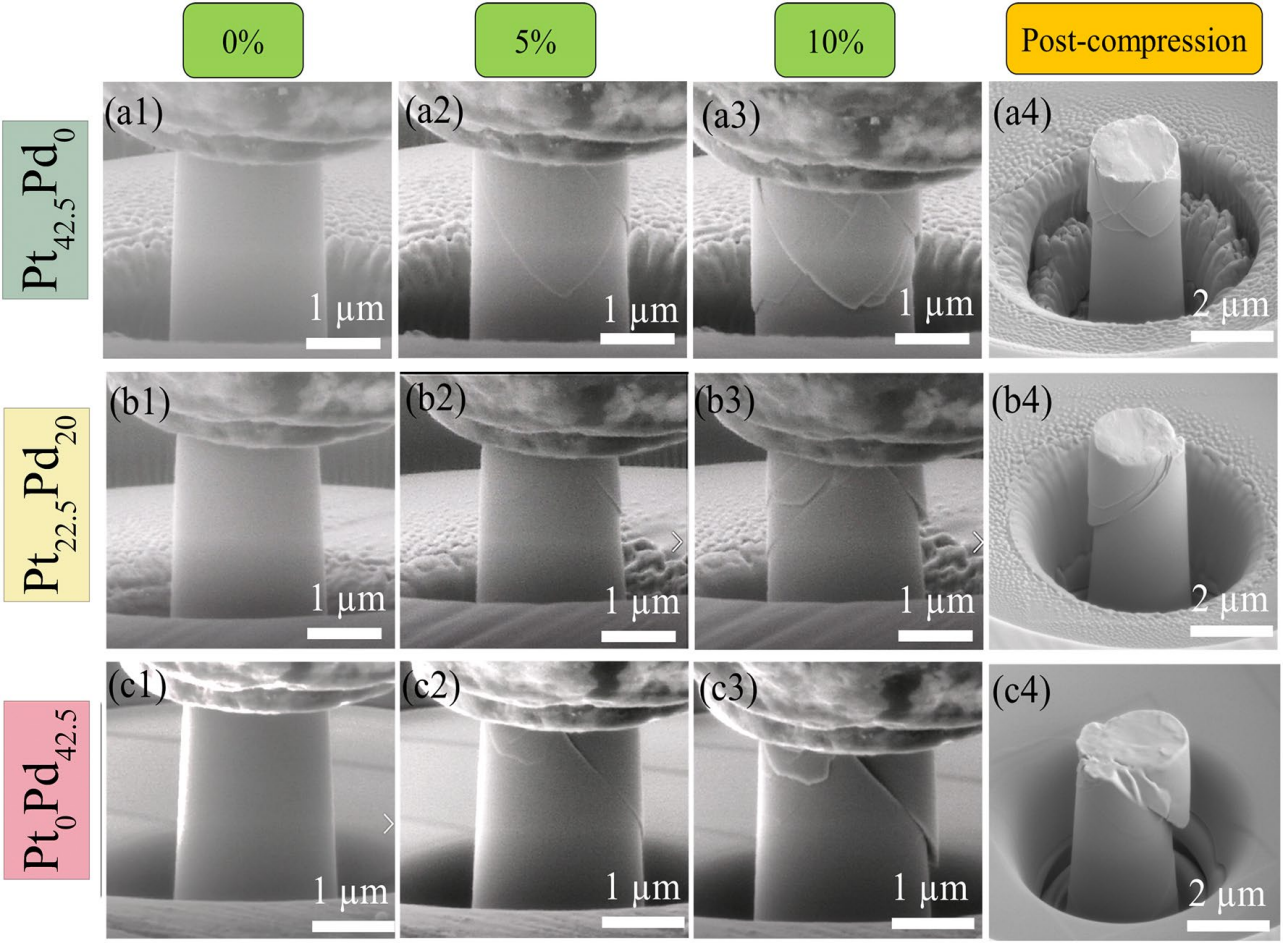
In-situ SEM image at strain of 0%, 5%, and 10% for micro-pillars of (**a1**–**a3**) Pt_42.5_Pd_0_, (**b1**–**b3**) Pt_22.5_Pd_20_, and (**c1**–**c3**) Pt_0_Pd_42.5_ amorphous alloys; post compression SEM images for (**a4**) Pt_42.5_Pd_0_, (**b4**) Pt_22.5_Pd_20_, and (**c4**) Pt_0_Pd_42.5_ alloys indicating multiple shear band formation for Pt_42.5_Pd_0_ in contrast to one major shear band for Pt_0_Pd_42.5_ alloy.

Engineering stress–strain response obtained from micro-pillar compression for Pt_42.5−x_Pd_x_Cu_27_Ni_9.5_P_21_ (x = 0, 20, and 42.5) alloys are shown in Fig. 4a–c. Stresses and strains were calculated with consideration of taper correction. At least three micro-pillars were tested for each alloy as shown. The experiments were stopped at a strain of ~ 10% to examine the post-deformation morphologies of the micropillars. For all three alloys, the stress initially increases linearly with strain followed by numerous serrations or stress drops. The stress drops were elastic-like with the slope of the straight portion in between the drops approximately equal to the slope of the initial elastic deformation. Elastic unloading before the elastic reloading process was due to the readjustment of the indenter position to ensure steady displacement rate. The average value of yield strength was determined from the stress at the first serration to be ~ 0.95 GPa, ~ 1.05 GPa, and ~ 1.25 GPa for Pt_42.5_Pd_0_, Pt_22.5_Pd_20_, and Pt_0_Pd_42.5_ alloys, respectively. The stress drops in stress–strain curves for all alloys were attributed to the nucleation and propagation of shear bands^56^. The amplitude of stress drops increased while its frequency decreased with increase in Pd content. In the insets of Fig. 4a–c, representative engineering stress–strain curves are shown to highlight the flow serrations while excluding the initial elastic loading segment (~ 2%). The size of stress drops (Δ*σ*) was measured from the stress–strain curves and the average value for each alloy is shown in Fig. 4d. The average magnitude of stress drop measured for Pt_42.5_Pd_0_ (~ 130 MPa) was ~ 7% and 35% lower in comparison to stress drops in Pt_22.5_Pd_20_ (~ 140 MPa) and Pt_0_Pd_42.5_ (~ 200 MPa), respectively. The magnitude of stress drop is an indirect measure of the tendency of the alloy towards stable or unstable shear band propagation. A smaller stress drop indicates relatively more stable deformation process^57^, in agreement with the observed homogeneous deformation behavior of Pt_42.5_Pd_0_ in nano-indentation experiments. In previous studies, brittle-natured Mg- and Au-based BMG micropillars exhibited smaller numbers of strain bursts during micro-compression in contrast to numerous strain bursts for ductile Zr-based BMG micropillars^58^. Individual serration events correspond to the accumulation and release of elastic energy in order to bypass the energy barrier for shear band formation^59^. The stored/released elastic energy within a single stress drop is calculated as^60^:

3 $$\Delta E = \frac{1}{2}\Delta \sigma \varepsilon_{e} \pi \left( \frac{d}{2} \right)^{2} h$$

where *d* and *h* are the diameter and height of the pillar (*h* = 2*d*) and $\varepsilon_{e}$ is the elastic strain. The area of the shear plane A may be calculated as A = π[d/(2sinθ)]^2^, where θ is the angle between shear plane and the loading axis. The elastic energy release for each shear plane is measured according to ΔE/A = ε_e_Δσdsin^2^θ and shown in Fig. 4d for the studied bulk metallic glasses. The stored elastic energy increased with increase in Pd content. More energy released during the stress drops may increase the local temperature leading to quicker shear band sliding and more localized deformation^9^. Further, the larger standard deviation in stored elastic energy, depicted by the larger separation between the solid lines in Fig. 4d, indicates more heterogeneous distribution of stored elastic energy in the Pd-rich alloys. In-situ SEM images of the micro-pillars at the strains of 0%, 5%, and 10% are shown in Fig. 5 a1–a3, b1–b3, c1–5c3 for Pt_42.5_Pd_0_, Pt_22.5_Pd_20_, and Pt_0_Pd_42.5_ BMGs, respectively. Figure 5a4, b4, c4 show the post-compression images for Pt_42.5_Pd_0_, Pt_22.5_Pd_20_, and Pt_0_Pd_42.5_ alloys, respectively. Multiple intersecting shear bands are seen for Pt_42.5_Pd_0_ micro-pillars during compression test (Fig. 5a1–a4) and pronounced interaction of the shear bands indicates more homogeneous plastic deformation for Pt_42.5_Pd_0_. The density of shear bands decreased for Pt_22.5_Pd_20_ (Fig. 5b1–b4) and the micro-pillars for Pt_0_Pd_42.5_ alloy failed primarily by a single major shear band (Fig. 5c1–c4), indicating highly localized deformation. Activation of multiple shear bands promotes plasticity accommodation in case of Pt_42.5_Pd_0_, resulting in overall higher ductility as compared to the Pd-rich alloys. Higher fractions of more closely spaced shear bands lead to greater plasticity in amorphous alloys as plastic flow may begin easily on preexisting shear bands leading to larger distribution of shear rather than catastrophic failure^54^. Also, the presence of more shear bands helps in dissipating the energy of primary shear bands during plastic deformation^61^. The number of serrations seen in the stress–strain curves in Fig. 5 is significantly higher compared to the number of observed shear bands in the SEM images of the micro-pillars, which may be attributed to the formation and propagation of new shear bands as well as the interaction, pinning, and reactivation of the preexisting ones^62^.

### High energy X-ray diffraction

**Figure 6:**
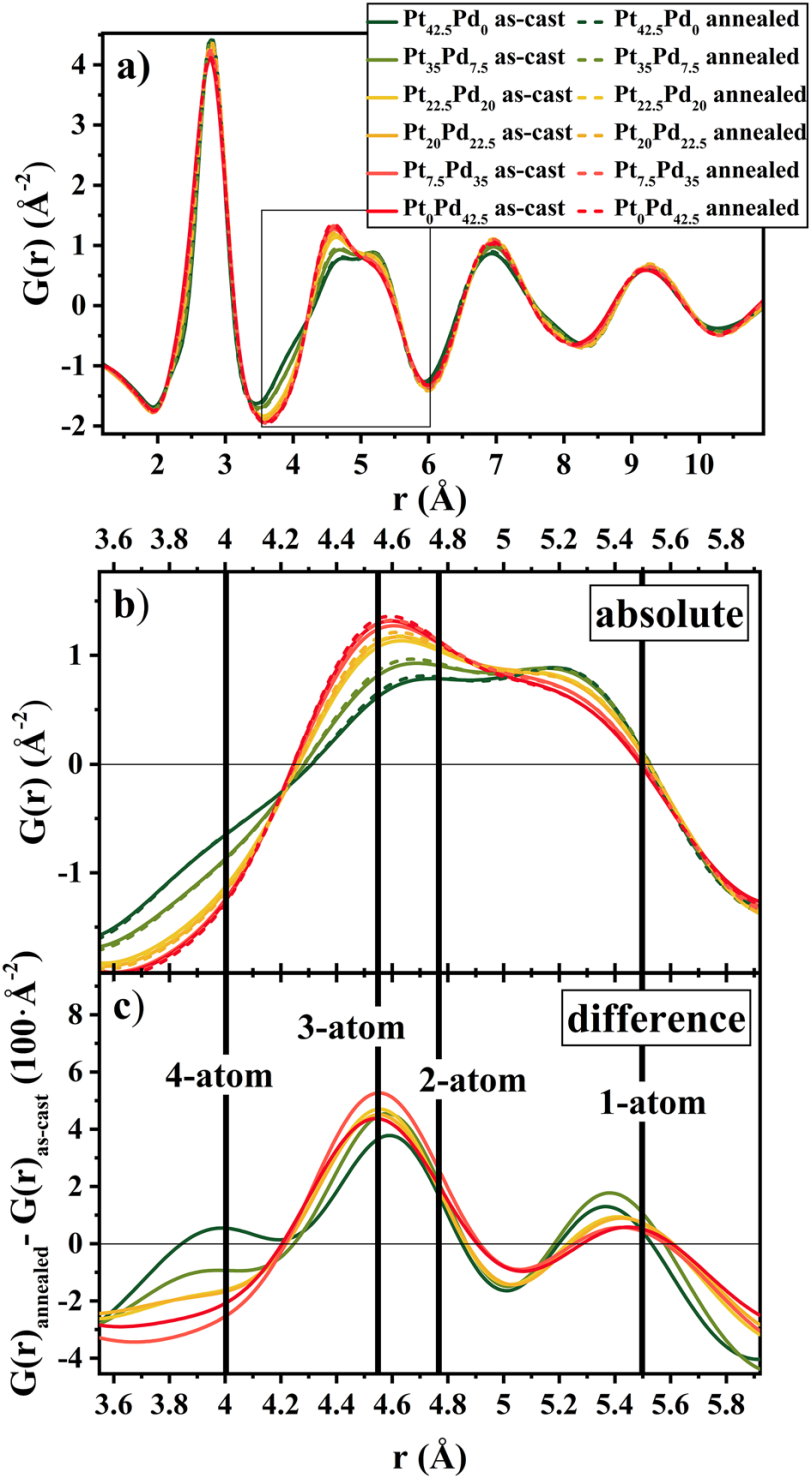
(**a**) The reduced pair distribution function G(*r*) for Pt_42.5−x_Pd_x_Cu_27_Ni_9.5_P_21_ BMGs, where x = 0, 7.5, 20, 22.5, 35, and 42.5 at 298 K in the as-cast state (full lines) and after annealing for 24 h at T_g_-50 K (dashed lines). (**b**) Magnified view of the box in a), featuring the second peak of G(r) for all alloys in annealed and as-cast state. The second peak of G(r) corresponds to the second nearest neighbor distances, therefore bearing information of the cluster connection schemes. (**c**) Difference in the reduced pair distribution function between annealed and as-cast state. A large change on the length scale of 4.55 Å is seen, corresponding to a distance of $\sqrt {8/3}$ r_1._ The vertical lines in (**b**) and (**c**) represent the most likely second nearest neighbor distances if adjacent clusters share one (2 r_1_), two ($\sqrt 2$ r_1_), three ($\sqrt {8/3}$ r_1_) or four atoms ($\sqrt 3$ r_1_).

Synchrotron diffraction experiments were performed for the annealed (T_g_-50 K for 24 h) and as-cast samples (5 mm rods) and the reduced pair-distribution function (PDF) for six chosen alloys are shown in Fig. 6a. The differences in structure with changing Pt/Pd content become evident similar to previous report^26^. The main difference is in the 2nd peak of G(r), which describes the 2nd coordination shell and provides information about the interconnectivity of clusters, namely how many atoms are shared by adjacent clusters^63^. Detailed justification on the structural differences as a function of Pd content that can be derived from diffraction data is discussed in prior work^26^. An enlarged view of this region is shown in Fig. 6b. The distances referring of the different cluster connection schemes are highlighted by vertical lines in the plot, marking the distances, where adjacent clusters share one (2 r_1_), two ($\sqrt 2$ r_1_), three ($\sqrt {8/3}$ r_1_) or four atoms ($\sqrt 3$ r_1_) ^63^. The width of the lines is used to account for the slight shift of r_1_ with composition. With increasing Pd-content, a peak at ~ 4.5 Å evolves, which is related to the 3-atom connection (meaning that adjacent clusters share 3 atoms), while the shoulder at 5.3 Å decreases.

Due to the small changes, a differential plot is used to compare the as-cast with the annealed state as shown in Fig. 6c. In the differential plot of Fig. 6c, the PDFs of the annealed samples (dashed lines) are more pronounced at the length scale corresponding to 3-atom connections. Further, annealing leads to a decrease in intensity of G(r) corresponding to the length scale of 4-atom connections, while the intensities on the spatial length of 2- and 1-atom connections remain rather unchanged.

## Discussion

Changes in composition by the gradual replacement of Pt with Pd atoms as well as thermal history showed a significant effect on the deformation behavior of Pt_42.5−x_Pd_x_Cu_27_Ni_9.5_P_21_ bulk metallic glasses. For the Pt-rich alloys, relatively more ductile behavior was observed, characterized by smoother load-depth curves in nanoindentation (Fig. 1), high value of SRS (Fig. 3), and multiple intersecting shear bands formed in micro-pillar compression tests (Fig. 5). In contrast, the Pd-rich alloys showed more brittle characteristics. Amorphous alloys in which plastic flow is driven by the activation of a small number of large shear bands show large values of *h*_discrete_/*h*_plastic_^20^. This suggests that Pd addition influences the nucleation and propagation of shear bands. Further, higher numbers of large displacement bursts (or pop-ins) for the alloys with higher Pd content correspond to greater shear displacement within the shear band and more localized deformation behavior^54,61^_._ Annealing led to pronounced increase in the discrete plasticity ratio (Fig. 2d) as well as the magnitude of displacement bursts for each alloy (Fig. 2b).

Relatively more ductile behavior of the Pt-rich alloys results from easier nucleation of smaller STZs, leading to more homogeneous plastic flow. Alloys with higher Pd content were characterized by lower strain rate sensitivity and higher STZ volume, indicating a propensity for brittle deformation.

A greater tendency towards cooling rate dependent embrittlement was previously described within the framework of critical fictive temperature for Pd_43_Cu_27_Ni_10_P_20_ BMG in comparison to Pt_57.5_Cu_14.7_Ni_5.3_P_22.5_ BMG^64^. However, a systematic study on the role of Pt and Pd is lacking. The current work provides quantitative assessment of the change in deformation behavior with the substitution of Pt by Pd for a set of alloys with the same stoichiometry.

Previous high energy synchrotron diffraction experiments suggested the presence of different dominant atomic clusters, trigonal prisms and icosahedrons, together with changing connection schemes and ratios within these clusters as a function of Pt and Pd content^26^. Simulations of Ding et al. showed that the different connection schemes respond differently to external strains, which can ultimately have a large effect on the mechanical behavior of the glass, when they rearrange with changes in composition or thermal history. Based on these simulations, 3-atom connections (face sharing), whose signature in G(r) increases with Pd content, are the only connecting scheme that leads to a smaller local elastic strain compared to the macroscopic deformation. In contrast, 1-atom connections (vertex sharing) show local elastic deformations similar to the macroscopic deformation, while 2-atom (edge sharing) and 4-atom connections (squashed tetrahedra sharing) show higher local elastic strain compared to the macroscopic strain. Ultimately this means that the face sharing 3-atom connections tend to form a relatively stiffer structure and can therefore, be associated with more brittle deformation behavior^63^. This supports the current experimental observations of brittle deformation behavior with increasing Pd-content, characterized by small strain rate sensitivity, more serrated flow, and more localized deformation^26^.

**Figure 7:**
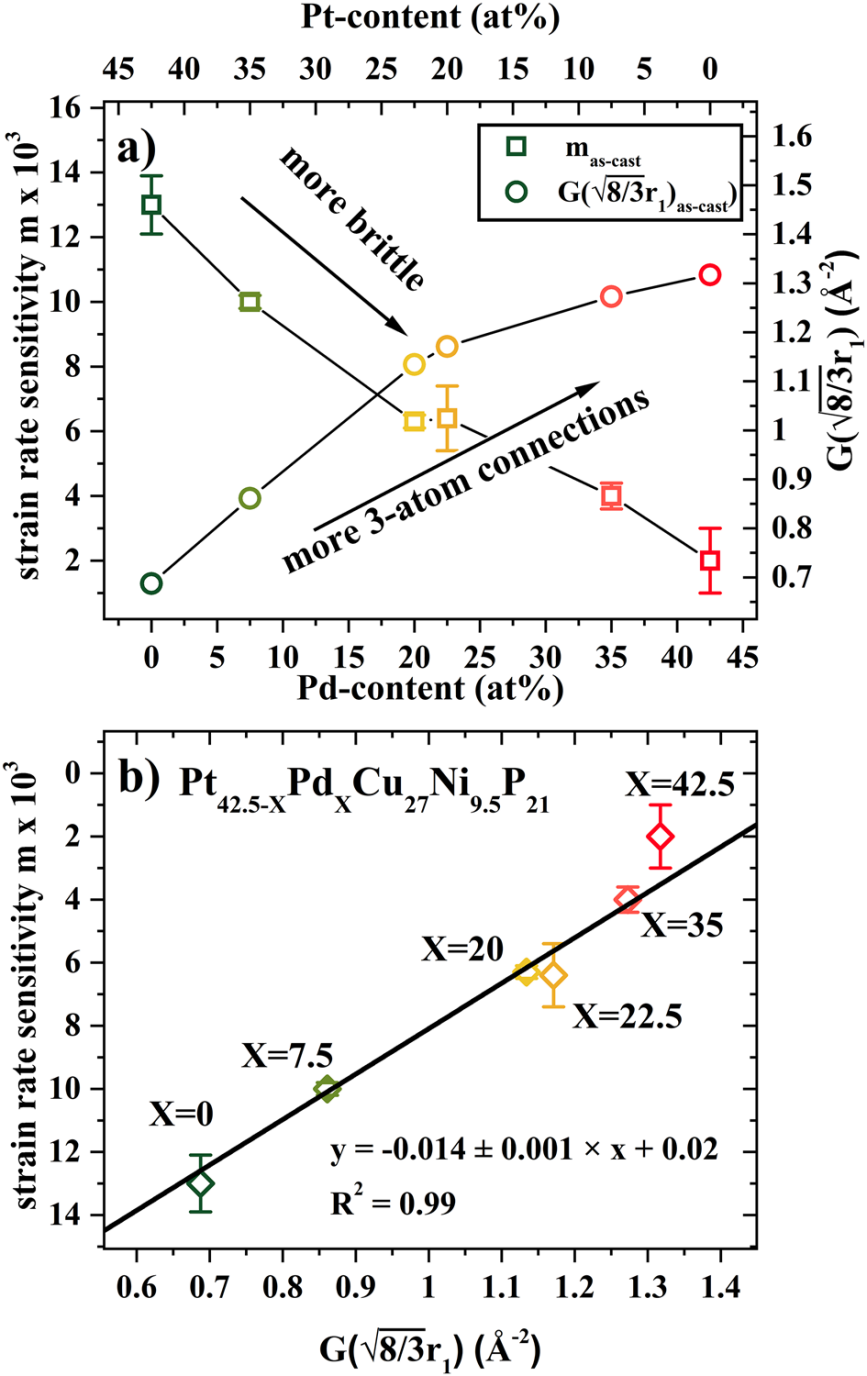
(**a**) Strain rate sensitivity m for as-cast state as a function of Pd-content (left axis, open squares) and absolute value of reduced pair distribution function at the length scale of 3-atom connections G($\sqrt {8/3}$ r_1_) in the as-cast state as a function of Pd-content (right axis, open circles). A decrease in SRS with increase in Pd content, associated with more brittle behavior, is observed. More rigid 3-atom connections in the reduced pair distribution function are observed with increase in Pd content, which is also characteristic of relatively brittle behavior. (**b**) Scatter plot of m_as-cast_ and G($\sqrt {8/3}$ r_1_)_as-cast_ showing the correlation of decreasing strain-rate sensitivity with increasing fraction of 3-atom cluster connections.

For a more quantitative analysis the structural features, namely the significance of 3-atom connection schemes of a specific composition in the series of interrelated alloys is correlated with its mechanical behavior in terms of SRS, with a high SRS indicative of more ductile behavior. Hence, the change in SRS with Pd content is directly compared with the value of PDF for 3-atom connections G(r = $\sqrt {8/3}$ r_1_), used to quantify the significance of 3-atom connections. The distribution of the connecting schemes may be described by a Gaussian function^65^. As a result, changes in intensity of G(r = $\sqrt {8/3}$ r_1_) may also be caused by broadening of the neighboring 2-atom and 4-atom connections. For this parameter to be significant, we assume no significant changes in the width of Gaussian distribution of each connecting scheme. Figure 7a shows a consistent decrease of SRS (m) with rising G(r = $\sqrt {8/3}$ r_1_). The increasing number of 3-atom connections with increasing Pd content may be attributed to a change in the dominant structural motifs occurring in the Pt-rich (trigonal prisms) and Pd-rich (icosahedra) subsystems. Even though, Pt and Pd are considered topologically equivalent in structural models, the differences in their electronic configurations (Pt: ([Xe]4f^14^5d^9^6s) and Pd: ([Kr]4d^10^)) as well as minor changes in their enthalpy of mixing with Ni (Pd–Ni 0 kJ, Pt-Ni-5 kJ (at equiatomic composition) might cause these changes in cluster distribution. Ultimately, this difference in chemistry of the topologically similar Pt and Pd atoms might lead to the different topology of the clusters and consequently their interconnection^66^. To further support our interpretation of increasing 3-atom connection with increasing Pd content and therefore increasing number of icosahedra, while decreasing the number of trigonal prisms one has to consider the geometry of these structural units. A perfect icosahedron has 20 triangular faces, which can be shared by adjacent clusters. Although, this may not be the exclusive connection scheme present in Pd-rich systems, the large number of triangular faces may lead to dominance of 3-atom connections. It has been suggested by Gaskell that trigonal prisms often connect via two atoms and 4 atoms, whereas 3-atom connections get less probable^67^. Ultimately, the replacement of Pd atoms by Pt atoms will lead to a change in the ratio between icosahedra and trigonal prisms and therefore alter the distribution of the connection schemes. These changes in the distribution of cluster connections are visible in the second peak of the reduced pair distribution function (Fig. 6). In summary, with increasing Pt content, the fraction of 3-atom connection decreases and the other connection schemes gain importance, which is a consequence of the reduction of the icosahedral SRO. A more detailed justification and discussion of the structural changes with Pt/Pd content is provided in our earlier work^26^ as well as its respective Peer Review File^66^. Figure 7b is showing the correlation between SRS and the frequency of finding an atom at the distance of the 3-atom connections G(r = $\sqrt {8/3}$ r_1_) (R^2^ equal to 0.99), clearly supports our working hypothesis of an increasing icosahedral SRO with increasing Pd-content resulting in a macroscopically and microscopically more brittle mechanical behavior.

**Figure 8:**
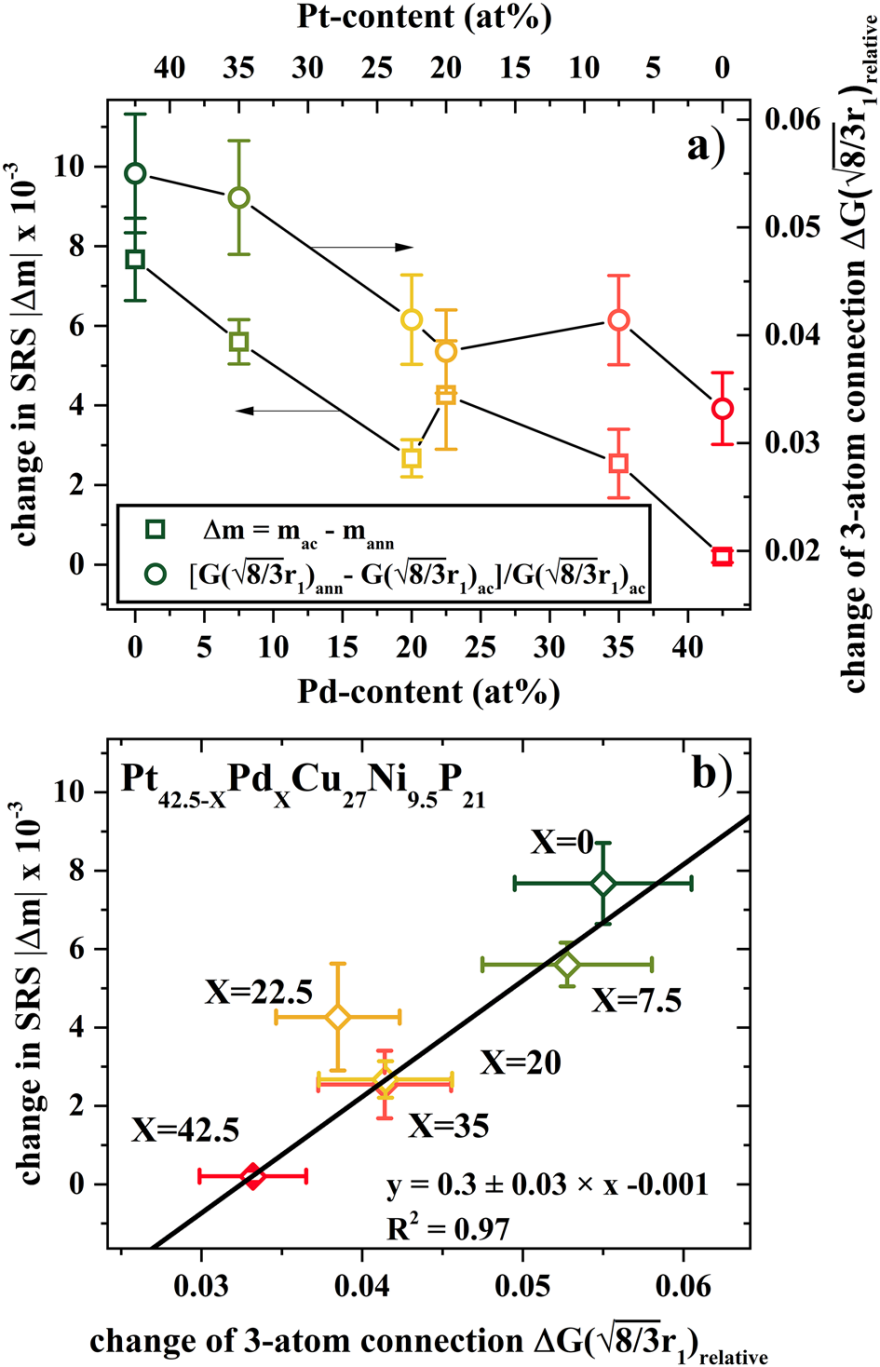
(**a**) Change in strain rate sensitivity (Δm) after annealing for 24 h at T_g_-50 K in reference to the as-cast state as a function of Pd-content (left axis, open squares) and relative change of reduced pair distribution function at the length scale of 3-atom connections ΔG($\sqrt {8/3}$ r_1_)_relative_ after annealing for 24 h at T_g_-50 K in reference to the as-cast state as a function of Pd-content (right axis, open circles). (**b**) Scatter plot of Δm and ΔG($\sqrt {8/3}$ r_1_)_relative_ showing the correlation of change in strain-rate sensitivity with increasing fraction of 3-atom cluster connections.

To quantify the structural changes from annealing, the difference in reduced PDF on the length scale of 3-atom connections, G($\sqrt {8/3}$ r_1_), is determined as function of Pd-content (Fig. 8). To account for the scaling of the different atomic form factors with composition, the changes are calculated with respect to their as-cast reference value G($\sqrt {8/3}$ r_1_)_ac_ leading to a relative change, G($\sqrt {8/3}$ r_1_)_relative_ = [ G($\sqrt {8/3}$ r_1_)_rel_ − G($\sqrt {8/3}$ r_1_)_ac_]/G($\sqrt {8/3}$ r_1_)_ac_.

The change in SRS (Δm) after the annealing (left axis) is shown relative to the change in reduced PDF (right axis) in Fig. 8a, both showing a similar trend with variation in Pd-content. For the initially ductile Pt-rich glasses, annealing leads to the highest relative increase in the intensity of G(r) at the length scale of 3-atom connections accompanied by the largest change in SRS. This indicates that the largest drop in ductility is mirrored by the largest relative change in the probability of atoms being located at the length scale, geometrically predicted, for 3-atom connections. Vice versa, the Pd-rich alloys show almost no change in their SRS, which is consistent with minor changes in G($\sqrt {8/3}$ r_1_). The scatter plot of Δm versus ΔG($\sqrt {8/3}$ r_1_)_relative_ in Fig. 8b shows the quantitative correlation between the changes in intensity at the length scale of G($\sqrt {8/3}$ r_1_), interpreted as the signature of 3-atom connections, and SRS, being used to describe the ductility of the material. Interestingly, the simulations of Ding et al. showed that structural ordering during cooling leads to an increase in the stiffer 3-atom connections^63^. To a certain degree isothermal annealing resembles the effects of slow cooling, as the system also undergoes structural relaxation and ordering to an enthalpically lower state leading to a lower fictive temperature. Therefore, further resemblance between experimental data and the reported simulations is given.

The experimental results indicate that the absolute structural changes facilitated by compositional variation, when platinum is systematically replaced by topologically equivalent palladium atoms, are much more significant than those due to annealing. In contrast, the absolute changes in ductility/SRS with composition or due to annealing are comparable in magnitude. This indicates, that while the distinctiveness of 3-atom connections might play a significant role in the mechanical properties of metallic glasses, they do not directly allow a prediction of ductility. Ultimately, the deformation process of multicomponent metallic glasses with respect to chemical and thermal changes are of course too complex to be solved by a single parameter. Still, this does not come as a surprise as there are with a probability bordering on certainty further (structural) mechanisms and effects in combination with the limitations of structural data of multi-component systems obtained in a total scattering diffraction experiment. In total scattering, the atomic pairs with the highest atomic form factor/strongest scattering ability, dominate the reduced pair distribution function, which is a superposition of all atomic pairs involved. For the investigated systems, this means that only noble-noble metal interactions are probed with minor contributions of noble metal-Cu and Cu-Cu partials, whereas any pair associated with P remains unaccounted. Still, for the Pt/Pd–Pt/Pd partials, the increase in 3-atom connections with Pd-content and with relaxation shows good quantitative agreement, which is in line with simulations. This work may stimulate further investigations on the relation between local structure and mechanical performance of metallic glasses.

## Conclusions

In summary, the plastic deformation mechanism of interrelated Pt_42.5−x_Pd_x_Cu_27_Ni_9.5_P_21_ bulk metallic glasses was studied, where platinum was systematically replaced by topologically equivalent palladium atoms. Increase in Pd content resulted in increasing hardness and yield strength and a drop in strain rate sensitivity. The Pt-rich bulk metallic glasses showed higher strain rate sensitivity, lower discrete plasticity ratio in nano-indentation experiments, smaller stress-drops, and multiple shear band formation in micro-pillar compression, indicating more homogeneous flow compared to the Pd-rich alloys. Similar to the addition of Pd, embrittling effects were observed after sub-T_g_ annealing (T_g_-50 K for 24 h) of the samples, both effects might be connected to a similarly increasing trend in the reduced pair distribution function on the length scale of rigid 3-atom cluster connections. This present systematic study helps to further shed light on the interrelation of structure and mechanical properties in metallic glasses through alloying and heat treatment.

## Materials and methods

### Sample preparation

Pt_42.5−x_Pd_x_Cu_27_Ni_9.5_P_21_ (x = 0 and 42.5) master alloys were synthesized by arc melting the mixture of pure elements (Pt, Pd, Cu, and Ni with purity of > 99.95%) under a Ti-gettered high purity argon atmosphere. Each ingot was flipped and re-melted for at least four times to ensure homogeneity in composition. Subsequently, the pre-alloys were placed on top of P in a fused quartz tube and heated inductively followed by a fluxing process in dehydrated B_2_O_3_ for at least 20 h at 1200 °C in a fused silica tube to remove impurities. Afterwards, the master-alloys are mixed in the ratio of the final composition; Pt_42.5−x_Pd_x_Cu_27_Ni_9.5_P_21_, where x is x = 0, 2.5, 7.5, 12.5, 17.5, 20, 22.5, 30, 35, 40, 42.5 at %. This solid mixture is then re-melted in an arc-melter under a Ti-gettered high-purity argon atmosphere to ensure a homogeneous sample.

Amorphous samples were prepared by inductive re-melting of the ingots and tilt- casting in a water-cooled copper mold with 5 mm diameter under argon atmosphere (Ar 6.0). Few select compositions with Pd content x = 0, 7.5, 20, 22.5, 35, 42.5 at % samples were annealed in a Perkin Elmer DSC 8000 at T_g_-50 K for a duration of 24 h under a high purity Ar (Ar 6.0) atmosphere. All samples, annealed and as-cast, were polished to mirror surface finish for nano-mechanical characterization. Chemical compositions of the alloys were confirmed using scanning electron microscopy (SEM, FEI, Hillsboro, OR, USA) equipped with energy dispersive spectroscopy (EDS).

### Nano-indentation

Nano-indentation was done using a TI-Premier Triboindenter (Bruker, Minneapolis, MN, USA) with a diamond Berkovich tip at room temperature, a peak load of 100 mN, and a loading and unloading rate of 20 mN/s. The hardness and modulus were determined using Oliver and Pharr method^68^. Strain rate sensitivity (SRS) was calculated by nano-indentation in displacement-control mode with applied strain rates of 4.0 × 10^–2^ s^−1^, 1.2 × 10^–1^ s^−1^, 4.0 × 10^–1^ s^−1^. An average of sixteen indents were performed to obtain the average and standard deviation. Distance between adjacent indents was more than 100 μm to avoid overlap of their plastic zones. All tests were performed in the center of the samples in order to avoid the influence of cooling rate on local structural state. Thermal drift rate was measured and maintained below 0.05 nm/s for all tests.

### Micro-pillar compression

For micro-pillar compression tests, pillars ~ 5 μm in height and ~ 2.5 μm in diameter were milled in select alloys, namely Pt_42.5−x_Pd_x_Cu_27_Ni_9.5_P_21_ (x = 0, 20, and 42.5), using FEI Nova NanoLab 200 FIB-SEM using Ga ion beam, with current ranging from 5 nA to 10 pA. The top and bottom diameter of the micro-pillars were measured, and the taper angle was determined to be ~ 2°, which was considered for further analysis. PI88 SEM Picoindenter (Bruker, Minneapolis, MN, USA) with a 5 µm diameter flat diamond punch was used for the micro-pillar compression tests. Tests were performed in displacement-control mode at a strain rate of 6 $\times$ 10^–3^ s^−1^_._ The recorded load versus displacement was converted to engineering stress–strain curves using the micro-pillar dimensions. A minimum of three micro-pillars were milled for each alloy to determine the standard deviation.

### High-energy synchrotron X-ray diffraction

High-energy synchrotron radiation experiments were performed at beamline P21.2 at PETRAIII at Deutsches Elektronensynchrotron (DESY) synchrotron facility. Measurements were carried out in transmission geometry at a radiation energy of 70 keV (λ = 0.1771 Å) with a beam size of 0.5 × 0.5 mm. Disc-shaped samples were cut from 5 mm rods and were irradiated in the middle of the sample. A VAREX XRD4343CT detector with a pixel size of 150 × 150 µm and a resolution of 2880 × 2880 pixels was used for the recording of the patterns with a summed exposure time of 5 s. For the measurements, a set of five pictures was averaged, leading to a summed total exposure time of 25 s. The two-dimensional X-ray diffraction patterns were integrated using pyFAI integrate. For further processing, like background subtraction, corrections for sample absorption, polarization and multiple scattering PDFgetX2 software was used^69^.

The total structure factor S(Q) was calculated as^70^

1 $$S\left( Q \right) = 1 + \frac{{I_{C} \left( Q \right) - f\left( Q \right)^{2} }}{{f\left( Q \right)^{2} }},$$

where I_C_(Q) is the coherently scattered intensity, f(Q) the atomic form factor, and Q is the scattering vector. The angle brackets denote a compositional average over all constituents.

To obtain the reduced pair distribution function, G(r), a Fourier transformation of the total structure factor leads to:

2 $$G\left( r \right) = \frac{2}{\pi }\mathop \int \limits_{0}^{\infty } Q\left[ {S\left( Q \right) - 1} \right]\sin \left( {Qr} \right)dQ,$$

where r is the distance to the reference atom. Each G(r) pattern was optimized using an optimization algorithm in PDFgetX2 as described by Wei et al.^71^ with a maximum Q-range (Q_max_) for the Fourier transformation of S(Q) of 15 Å^−1^. This value is sufficient to obtain the needed degree of details in G(r) for metallic glasses, as shown in prior studies^26,72^.

## Supplementary Information

Supplementary Information 1.

## Data Availability

The datasets generated during and/or analyzed during the current study are available from the corresponding authors on reasonable request.
