# Supplementary material for: Effect of composition and thermal history on deformation behavior and cluster connections in model bulk metallic glasses
Source: Sci Rep. 2022 Oct 12;12:17133. doi: 10.1038/s41598-022-20938-6 (PMC9556669; doi:10.1038/s41598-022-20938-6)
Supplement: Supplementary file 1 — Supplementary Information 1. [file 41598_2022_20938_MOESM1_ESM.docx]

**Supplementary Information**





**Figure SI 1.** Hardness as a function of the strain rate on a double-logarithmic scale, obtained from nano-indentation for as-cast (a) samples and annealed samples (b)) with the Pt_42.5-x_Pd_x_Cu_27_Ni_9.5_P_21_ composition for different amounts of Pd. Linear fits on the double-logarithm scale are used to determine the strain-rate sensitivity m.
